# Rest but busy: Aberrant resting‐state functional connectivity of triple network model in insomnia

**DOI:** 10.1002/brb3.876

**Published:** 2018-01-04

**Authors:** Xiaojuan Dong, Haixia Qin, Taoyu Wu, Hua Hu, Keren Liao, Fei Cheng, Dong Gao, Xu Lei

**Affiliations:** ^1^ Sleep and NeuroImaging Center Faculty of Psychology Southwest University Chongqing China; ^2^ Key Laboratory of Cognition and Personality of Ministry of Education Chongqing China; ^3^ School of Psychological and Cognitive Sciences PKU‐IDG/McGovern Institute for Brain Research Beijing Key Laboratory of Behavior and Mental Health Peking University Beijing China; ^4^ Department of Psychiatry the First Affiliated Hospital of Chongqing Medical University Chongqing China; ^5^ Sleep Psychology Center Daping Hospital Third Military Medical University Chongqing China

**Keywords:** hyperarousal, insomnia disorder, large‐scale brain networks, resting‐state fMRI

## Abstract

**Introduction:**

One classical hypothesis among many models to explain the etiology and maintenance of insomnia disorder (ID) is hyperarousal. Aberrant functional connectivity among resting‐state large‐scale brain networks may be the underlying neurological mechanisms of this hypothesis. The aim of current study was to investigate the functional network connectivity (FNC) among large‐scale brain networks in patients with insomnia disorder (ID) during resting state.

**Methods:**

In the present study, the resting‐state fMRI was used to evaluate whether patients with ID showed aberrant FNC among dorsal attention network (DAN), frontoparietal control network (FPC), anterior default mode network (aDMN), and posterior default mode network (pDMN) compared with healthy good sleepers (HGSs). The Pearson's correlation analysis was employed to explore whether the abnormal FNC observed in patients with ID was associated with sleep parameters, cognitive and emotional scores, and behavioral performance assessed by questionnaires and tasks.

**Results:**

Patients with ID had worse subjective thought control ability measured by Thought Control Ability Questionnaire (TCAQ) and more negative affect than HGSs. Intriguingly, relative to HGSs, patients with ID showed a significant increase in FNC between DAN and FPC, but a significant decrease in FNC between aDMN and pDMN. Exploratory analysis in patients with ID revealed a significantly positive correlation between the DAN‐FPC FNC and reaction time (RT) of psychomotor vigilance task (PVT).

**Conclusion:**

The current study demonstrated that even during the resting state, the task‐activated and task‐deactivated large‐scale brain networks in insomniacs may still maintain a hyperarousal state, looking quite similar to the pattern in a task condition with external stimuli. Those results support the hyperarousal model of insomnia.

## 1 INTRODUCTION

Insomnia disorder (ID) is a prevalent clinical condition characterized by difficulty in falling asleep at bedtime, frequent awakenings in the middle of the night, waking up too early in the morning, and dissatisfaction with sleep duration and quality, often accompanying diminished memory, attention, academic performance, and deteriorated emotions including fatigue, decreased mood, irritability, or general malaise (Morin & Benca, 2012; Morin et al., 2015; Sateia, 2014). It has been considered as an acute disorder that is secondary to other psychotic/medical pathology such as depression, posttraumatic stress disorder, alcoholism, generalized anxiety, and obsessive compulsive disorder (Harvey & Tang, 2012; Kraus & Rabin, 2012).

Multiple hypotheses to explain ID have been proposed, such as cognitive, physiological, and cortical arousal and dysfunction in neuronal circuitry (Espie, 2002; Fortier‐Brochu, Beaulieu‐Bonneau, Ivers, & Morin, 2012), among which the most compelling is hyperarousal model. According to the hyperarousal hypothesis, insomniacs commonly show increased glucose metabolism in multiple brain areas (Nofzinger et al., 2004), elevated energy requirements in gray matter areas (Harper et al., 2013), or altered functional connectivity between brain regions (Li et al., 2017). Nofzinger et al. (2004) found that patients with primary insomnia had a small reduction of metabolism from wakefulness to NREM sleep in regions associated with cognition and emotion, including the amygdala, hypothalamus, thalamus, hippocampus, anterior cingulate cortex (ACC), medial prefrontal cortex (mPFC), and insular cortices (Nofzinger et al., 2004). Furthermore, a lack of deactivation of the default mode network (DMN) in insomniacs indicated a failure to disengage task‐irrelevant processing (Altena et al., 2008; Drummond et al., 2013).

Resting‐state fMRI studies of insomnia disorder have highlighted the importance of the salience network in hyperarousal and affective symptoms (Khazaie et al., 2017). Specifically, researches revealed decreased functional connectivity between the amygdala and insula, striatum, thalamus, and bilateral ACC, and increased functional connectivity between the amygdala and premotor cortex, sensorimotor cortex in patients with primary insomnia, suggesting a potential neurological mechanism for dysfunction of emotional control and affective disorder in insomniacs (Huang et al., 2012; Pace‐Schott et al., 2017). Findings from another study showed that activation of the insula within the salience network was increased in insomniacs, which could be indicative of subthreshold anxiety, worry, and rumination (Chen, Chang, Glover, & Gotlib, 2014). Previous studies of insomnia had an inconsistent conclusion on the functional connectivity within the DMN. In one study, insomniacs showed a significantly decreased functional connectivity between the medial prefrontal cortex (mPFC) and the right medial temporal lobe (rMTL), and also between the left MTL and the left inferior parietal cortices (Dai et al., 2015), while another study suggested stronger functional connectivity between retrosplenial cortex/hippocampus and various nodes of DMN in patients with ID (Regen et al., 2016). Moreover, it appeared to alter the functional connectivity between parietal and prefrontal cortices in insomniacs, inducing memory retrieval deficits (Li et al., 2014). However, most of these studies only focused on specific regions within one brain network. The functional connectivity between large‐scale brain networks in patients with ID remains unclear. Accordingly, our study aims to investigate the hyperarousal hypothesis of insomnia at the level of large‐scale brain network using independent component analysis (ICA), a data‐driven method, providing a new perspective for better understanding of the neural mechanism of insomnia.

The current study explored: (1) whether patients with ID showed aberrant functional connectivity among the large‐scale brain networks during resting state. We selected the anterior default mode network (aDMN) and posterior default mode network (pDMN) as the task‐deactivated networks, and dorsal attention network (DAN) and frontoparietal control network (FPC) as the task‐activated networks to evaluate the temporal relationship among them, that is, the functional network connectivity (FNC) (Jafri, Pearlson, Stevens, & Calhoun, 2008); (2) whether significant correlation was found in patients with ID between abnormal FNC with sleep parameters, cognitive and emotional scores, and reaction time (RT) of psychomotor vigilance task (PVT).

## 2 METHODS

### 2.1 Participants

Thirty‐one patients with ID and 32 age‐ and sex‐matched healthy good sleepers (HGSs) participated in this study. Patients were recruited from the Sleep Psychology Center of Daping Hospital, Third Military Medical University and diagnosed by experienced hospital psychiatrists (D.G. and H.H.). Twenty‐two patients (11 females and 11 males) were the first‐time visitors and had not ever taken medications, while the other nine patients had taken hypnotic medications or psychoactive medications before. The duration of taking medication ranged from 3 months to 3 years. The patients met the relevant diagnostic criteria of chronic insomnia disorder according to the International Classification of Sleep Disorders: Diagnostic and Coding Manual, 3rd ed. Specially, patients reported dissatisfaction with sleep characterized by either difficulty in initiating or maintaining sleep or early morning awakenings. The symptom of insomnia has lasted at least three nights a week for more than 3 months. HGSs were recruited from the same community via a newspaper advertisement and paid for participating in our experiment. All HGSs met the following criteria: a good sleep habit and a good sleep onset and/or maintenance; a regular dietary habit; no consumption of any stimulants, medications, alcohol, and cocaine for at least 3 months before the study; no history of neurological or psychological disorders; lower scores of Pittsburg Sleep Quality Index (PSQI) than 5. Patients with any findings of pathological brain MRI were excluded in the study. All subjects were given written informed consent prior to inclusion in the study. The study protocol was approved by the Ethics Committee of Third Military Medical University, China, and all procedures involved were in accordance with the Declaration of Helsinki.

### 2.2 Assessment

All participants were required to fill in questionnaires to measure sleep, insomnia severity, psychiatric characteristics, and perform the PVT to measure the daytime alertness and the sustained attention. Specifically, the following self‐report measurements and tasks were used:

1. The basic information of life questionnaire. The questionnaire collected the basic information of participants, involving the height, body weight, degree of education, present and historical clinical disease condition. as well as the medication compliance.
2. Pittsburg Sleep Quality Index (PSQI) (Buysse, Reynolds, Monk, Berman, & Kupfer, 1989). A widely used sleep questionnaire designed to assess different dimensions of sleep quality and quantity in the last month.
3. Insomnia Severity Index (ISI) (Bastien, Vallières, & Morin, 2001). A brief questionnaire of seven items designed to evaluate the severity of the nighttime and daytime symptoms of insomnia in the last 2 weeks prior to compilation.
4. Self‐Rating Depression Scale (SDS) (Zung, 1965). This 20‐item questionnaire measures the subjective severity of symptoms of depression.
5. Self‐Rating Anxiety Scale (SAS) (Zung, 1971). This 20‐item questionnaire measures the subjective severity of symptoms of anxiety.
6. Positive Affect and Negative Affect Schedule (PANAS) (Watson & Clark, 1988). Participants reported how often they had experienced 10 positive emotions (interested, excited, strong, enthusiastic, proud, alert, inspired, determined, attentive, and active) and 10 negative emotions (distressed, upset, guilty, scared, hostile, irritable, ashamed, nervous, jittery, and afraid) using a 5‐point rating scale (1 = *none of the time*, 2 = *a little of the time*, 3 = *some of the time*, 4 = *most of the time*, 5 = *all of the time*). Items were averaged to create subscale scores for daily positive and negative affects.
7. Thought Control Ability Questionnaire (TCAQ) (Wells & Davies, 1994). The 25‐item questionnaire measures the subjective thought control ability of participants before the sleep and daytime, such as “I often have difficulty falling asleep because I think of something about myself.”
8. Psychomotor Vigilance Task (PVT) (Drummond et al., 2005). The task can reflect the daytime alertness and the sustained attention of individuals. It has been proved to be highly sensitive to sleep lose. Participants were instructed to press a button as quickly as possible when a yellow millisecond‐counter appeared on a red small frame. Upon pressing the button, the red small frame became yellow and the screen displayed RT, giving an instant feedback on the individual's performance. Stimuli appeared in a random pattern with interstimuli interval varying between 2 and 10 s. The task lasted 5 min and consisted of approximately 40 stimulus presentations. Before the first trial, each participant had a 1‐minute habituation task. Finally, we calculated the median of RT as the PVT performance because some subjects had extreme RT trials.

### 2.3 Acquisition of resting‐state fMRI

Imaging data were acquired using a 3‐Tesla scanner (Magnetom TIM‐Trio, Siemens, Erlangen, Germany). Head movement was minimized by a cushioned head fixation device, and earplugs were used to attenuate scanner noise. Scanning was taken place from 2:00 to 5:00 p.m. Participants were instructed to fix on a cross in the center of black background screen without thinking intentionally in the mind and keep as motionless as possible. High‐resolution T1‐weighted anatomical images were collected using 3D spoiled gradient recalled (3DSPGR) sequence. Parameters were set such that TR/TE = 8.5/3.4 ms, flip angle = 12°, matrix = 512 × 512, field of view (FOV) = 240 × 240 mm^2^, with a voxel size of 1 mm^3^, 176 slices, 1 mm thick. Subsequently, functional images were collected using an echo‐planar imaging (EPI) sequence with the following parameters: TR/TE = 1500/29 ms, flip angle = 90°, matrix = 64 × 64, voxel size = 3 mm^3^, FOV = 192 × 192 mm^2^, axial slices = 25, thickness/gap = 5/0.5 mm. A total of 204 volumes were collected for the fMRI analysis and the first four volumes were discarded to ensure steady‐state longitudinal magnetization.

### 2.4 Resting‐state fMRI analysis

The Statistical Parametric Mapping software package, SPM8 RRID:SCR_007037 (http://www.fil.ion.ucl.ac.uk/spm/, Welcome Department of Cognitive Neurology, UK) was used to preprocess the imaging data. After discarding the first four volumes, the images were then corrected for the time delay between slices and the motion movement between volumes. One patient and two HGSs whose head movement was >1.5 mm were excluded. Spatial normalization was performed on the resulting images according to the standard Montreal Neurologic Institute (MNI) EPI template, resampled into a 3 × 3 × 3 mm^3^ voxel size. To reduce the effects of low‐frequency drift and high‐frequency noise, the data were processed to remove linear trends and filtered with 0.01–0.08 Hz, followed by spatial smoothing with FWHM = 6 mm. Six head motion parameters, six first derivatives, and 12 corresponding squared items (i.e., Friston‐24) were regressed from the resulting images. In addition, the voxel‐based morphometry (VBM RRID:SCR_014196) analysis was performed to decide whether participants have brain atrophy. The results showed that there was no brain atrophy of all subjects.

**Figure 1:**
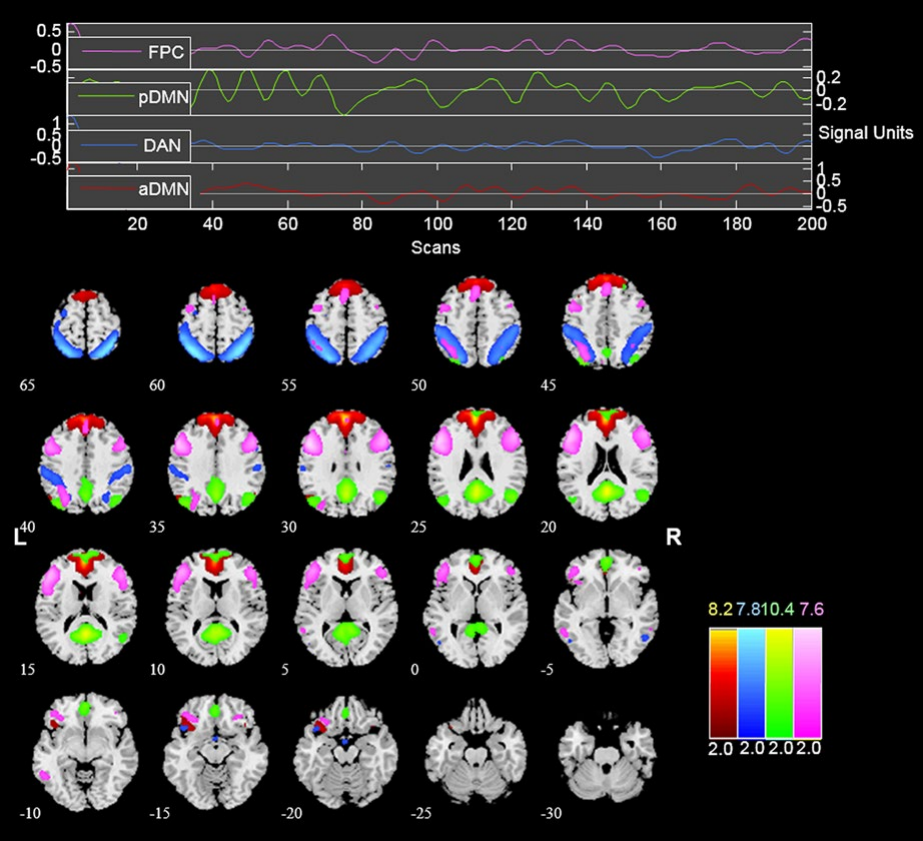
The spatial distribution of aDMN (red), DAN (blue), pDMN (green), and FPC (pink). Brain areas with intensities of 2 *SD* greater than the mean are shown. aDMN, anterior default mode network; DAN, dorsal attention network; pDMN, posterior default mode network; FPC, frontoparietal control network

After fMRI data preprocessing, the group independent component analysis (GICA) was performed using the GIFT RRID:SCR_001953 software (http://icatb.sourceforge.net/) (Calhoun, Adali, Pearlson, & Pekar, 2001) to retrieve resting‐state networks and subsequently to identify networks of interest. Preprocessed data from all subjects were submitted to the GIFT. The optimal number of components was set to 25, which was estimated using the minimum description length criterion (Li, Adalı, & Calhoun, 2007). After data reduction by principal component analysis (PCA), ICA decomposition was performed on concatenated datasets using the Extended Infomax algorithm. Individual time courses and components for each subject were back‐reconstructed, and the mean spatial maps for each group were transformed to *z*‐score for display purposes. We employed the anterior default mode network (aDMN), posterior default mode network (pDMN), dorsal attention network (DAN), and frontoparietal control network (FPC) maps from one of our previous resting‐state fMRI studies as spatial templates for component classification (Lei, Zhao, & Chen, 2013). The selected neural network corresponded to a component if it had the largest spatial correlation with the template and the correlation value was at least double than that of other networks. The spatial anatomy of the four networks were presented in Figure 1. The anatomical locations and the corresponding Montreal Neurological Institute (MNI) template space coordinates (Tzourio‐Mazoyer et al., 2002) of the brain regions were summarized in Table 1.

**Table 1:** Peak foci for the aDMN, pDMN, DAN, and FPC defined by group ICA

Regions	MNI coordinates			*T*
	*x*	*y*	*z*	
aDMN				
L medial prefrontal cortex	−3	45	18	22.53
L inferior frontal cortex	−33	18	−18	13.69
R middle frontal gyrus	42	36	−12	8.77
L middle temporal gyrus	−57	−18	−18	6.96
R precentral gyrus	54	−9	33	7.04
L precuneus	−18	−90	33	9.02
L angular	−48	−66	30	8.28
pDMN				
Medial frontal gyrus	0	57	−6	10.67
Posterior cingulate	9	−57	12	30.42
L precuneus	−39	−72	36	13.62
R middle temporal gyrus	48	−66	24	14.24
L superior frontal gyrus	−27	24	51	7.20
L precentral gyrus	−57	−3	12	5.15
DAN				
R inferior frontal gyrus	45	21	−12	7.14
L inferior frontal gyrus	−39	21	−18	6.53
L middle occipital gyrus	−48	−66	−9	9.57
R Lingual gyrus	9	−63	−3	6.04
R inferior temporal gyrus	54	−63	−6	7.26
R posterior parietal cortex	39	−45	51	19.46
R inferior frontal gyrus	57	6	36	9.31
FPC				
L inferior frontal gyrus	−48	42	6	19.82
R dorsolateral prefrontal gyrus	51	24	24	14.54
R middle temporal gyrus	63	−48	−3	8.37
Medial frontal gyrus	0	33	48	12.80
R cingulate gyrus	3	−39	36	6.61
R posterior parietal cortex	36	−72	39	6.32
R posterior cingulate	12	−57	9	7.48

aDMN, anterior default mode network; pDMN, posterior default mode network; DAN, dorsal attention network; FPC, frontoparietal control network; L, left hemisphere; R, right hemisphere. The significance threshold was set to *p *< .001, FDR‐corrected, with a minimum cluster size equal to 100 adjacent voxels.

After ICA analysis, we calculated the constrained minimal lagged correlation using the extension of GIFT RRID:SCR_001953 which is the functional network connectivity toolbox (FNC RRID:SCR_015731, http://mialab.mrn.org/software/#fnc) (Jafri et al., 2008; Xin & Lei, 2015) to measure the interactions of the four networks. The correlation among the time courses of the four networks was computed across each subject. After the Fisher Z translation of correlation coefficient, the significant temporal interactions between pDMN and aDMN and between DAN and FPC were examined using the two‐tailed two‐sample *t*‐tests.

### 2.5 Pearson's correlation analysis

The Pearson's correlation analysis was performed between the functional connectivity which was altered in patients with ID and sleep parameters, emotional and cognitive scores, and RT of PVT after controlling for the influence of age, gender, and education level. The multiple comparisons were corrected by using Bonferroni procedure in the current study.

## 3 RESULTS

### 3.1 Sample descriptives

Sample descriptives were summarized in Table 2. Two groups did not differ in age (*t*_58_ = 0.944, *p = *.349), gender (χ^2^ *= *0.00, *p = *1.00), and education level (*t*_58_ *= *−1.252, *p = *.216). Compared with HGSs, patients with ID showed significantly higher PSQI scores (*p *<* *.001), longer sleep onset latency (*p *<* *.001), lower sleep efficiency (*p *<* *.001), higher insomnia severity index (*p *<* *.001), and more subjective negative emotions measured by Self‐Rating Depression Scale (*t*_58_ *= *4.766, *p *<* *.001) and Self‐Rating Anxiety Scale (*t*_58_ *= *7.230, *p *<* *.001). In addition, there was a significant difference in RT of PVT (*t*_58_ *= *3.506, *p *=* *.001) between the two groups.

**Table 2:** Demographics, clinical data, and PVT performance of patients with insomnia disorder and healthy good sleepers

Characteristics	Patients with ID (*n *= 30)	HGSs (*n *= 30)	*p*
Age (years)	44.86 ± 11.62	42.03 ± 11.62	.349
Gender (male/female)	14/16	14/16	1.000
Education (years)	11.67 ± 2.70	12.60 ± 3.07	.216
Insomnia type	4.37 ± 1.47	—	
PSQI score	13.90 ± 3.74	3.47 ± 2.11	<.001
Sleep onset latency	49.33 ± 30.65	20.87 ± 10.00	<.001
Sleep efficiency (%)	61.12 ± 18.08	86.04 ± 9.56	<.001
ISI	17.07 ± 3.86	1.87 ± 1.59	<.001
SDS	51.89 ± 9.68	38.56 ± 11.88	<.001
SAS	51.42 ± 10.19	34.78 ± 7.42	<.001
PANAS: PA	25.00 ± 6.35	—	
PANAS: NA	21.50 ± 6.05	—	
TCAQ	72.37 ± 12.12	—	
PVT reaction time (seconds)	371.30 ± 52.50	328.47 ± 41.50	.001

ID, insomnia disorder; HGSs, healthy good sleepers; insomnia type (1 indicates <1 month; 2 indicates more than 1 month but <3 months; 3 indicates between 3 months and 6 months; 4 indicates between 6 months and 1 year; 5 indicates between 1 year and 3 years; 6 indicates more than 3 years); —, not applicable; PSQI, Pittsburgh Sleep Quality Index; ISI, Insomnia Severity Index; SDS, Self‐Rating Depression Scale; SAS, Self‐Rating Anxiety Scale; PANAS, Positive Affect and Negative Affect Scale; PA, positive affect; NA, negative affect; TCAQ, Thought Control Ability Questionnaire; PVT, psychomotor vigilance task.

The correlation analysis revealed that patients with ID who had higher PSQI scores showed worse thought control ability (*r *= −0.392, *p *=* *.039) measured by Thought Control Ability Questionnaire (TCAQ) and more negative affect (*r *= 0.518, *p *=* *.006) measured by Positive Affect and Negative Affect Schedule (PANAS).

### 3.2 Aberrant FNC in patients with ID

**Figure 2:**
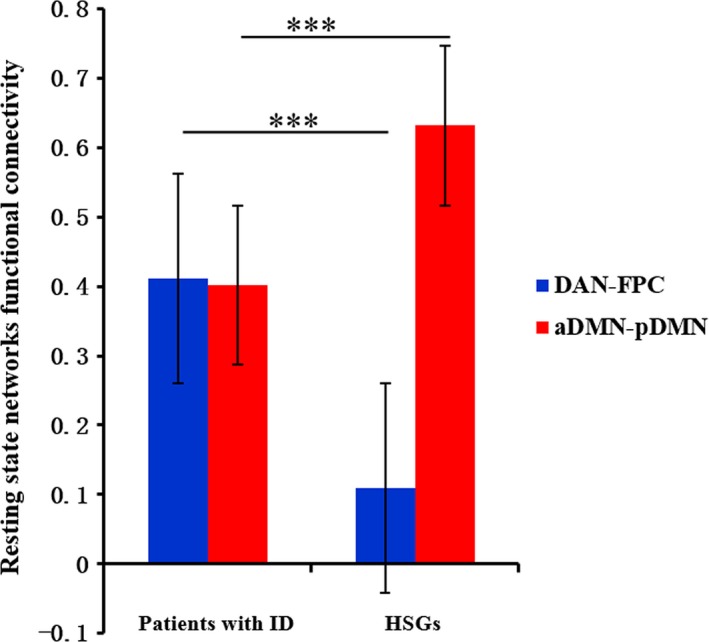
DAN‐FPC and aDMN‐pDMN functional connectivity of two groups. A significant difference in aDMN‐pDMN and DAN‐FPC connectivity strength between the two groups. ***p < .001

Patients with ID showed significantly increased functional connectivity between FPC and DAN (HGSs: 0.108, patients: 0.411; *t*_50.147_ *= *4.731, *p *<* *.001) and decreased functional connectivity between aDMN and pDMN (HGSs: 0.632, patients: 0.402; *t*_58_ *= *−3.851, *p *<* *.001), in comparison to HGSs (Figure 2).

### 3.3 Correlation results

**Figure 3:**
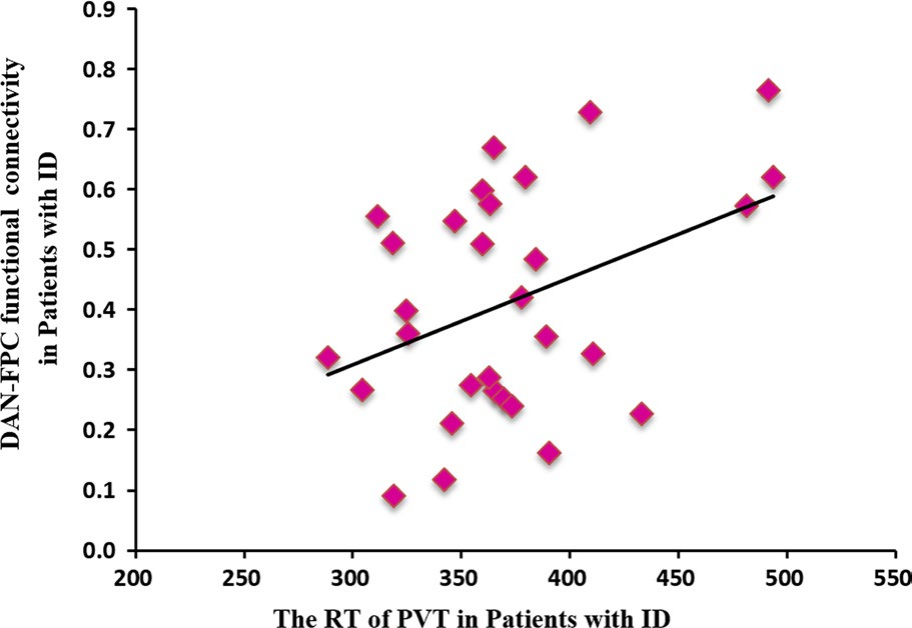
The functional connectivity between DAN and FPC and its relationship with the reaction time (RT) of psychomotor vigilance task (PVT) in patients with ID. Patients who have more increased functional connectivity between DAN and FPC were found to have worse PVT performance (*r *= 0.400, *p = *.035 < .05). Each magenta diamond represents a single subject

There was a significantly positive correlation between DAN‐FPC functional connectivity and RT of PVT (*r *= 0.400, *p = *.035) in patients with ID (Figure 3).

## 4 DISCUSSION

The current study investigated the aberrant temporal relationship among the resting‐state brain functional networks in patients with ID. Three main findings were observed. First, patients with ID exhibited a significant increase in DAN‐FPC functional connectivity. Second, a significant difference in RT of PVT was found between two groups and the PVT performance was significantly associated with DAN‐FPC functional connectivity in patients but not in HGSs. Finally, results revealed a significantly decreased functional connectivity between aDMN and pDMN in patients compared with the HGSs.

Behavioral performance and scale scores analysis revealed that patients with ID who had poorer subjective sleep quality measured by PSQI showed worse subjective thought control ability and more negative affect. Previous studies have demonstrated that the higher frequencies of mind wandering, daydream, and negative thought intrusion were associated with poorer sleep quality, in particular with poorer subjective sleep quality (Baker, Baldwin, & Garner, 2015; Carciofo, Du, Song, & Zhang, 2014). Worrisome thought is a center feature of several models of insomnia and involves persistent intrusive thoughts, which may impair the cognitive control in insomnia patients (Borkovec, Robinson, Pruzinsky, & DePree, 1983; Harvey, 2002; Lundh & Broman, 2000). These characteristics led insomniacs to present excessively negative cognitive activity, which caused hyperarousal and emotional distress (Harvey, 2002). Hence, it may declare that improving sleep quality contributes to enhancing subjective thought control ability and reducing the negative affect in insomniacs. Unexpectedly, a significantly positive correlation between positive affect and RT of PVT was found in insomniacs. Due to the hyperarousal in cognition, the clinical insomniacs may misunderstand these positive adjectives of the positive affect and negative affect scale (PANAS), which easily caused overactive positive affect that was adverse to behavioral performance.

In the current study, we found that the DAN‐FPC functional connectivity was significantly increased in insomniacs and this connectivity was significantly correlated with PVT performance. DAN and FPC are known as task‐activated networks. DAN includes the intraparietal sulcus (IPS) and the junction of the precentral and superior frontal sulcus (frontal eye field, FEF) in each hemisphere. Increasing evidence has highlighted that DAN modulates the goal‐directed attention processing and shows increased activity after presenting cues about where, when, or to what features direct their attention (Fox, Corbetta, Snyder, Vincent, & Raichle, 2006; Lei, Wang, Yuan, & Mantini, 2014). FPC, distributed in the frontoparietal areas, is typically engaged in externally directed tasks and has been implicated in the management of exogenous cognitive functions (Cole & Schneider, 2007; Liang, Zou, He, & Yang, 2016; Vincent, Kahn, Snyder, Raichle, & Buckner, 2008). A previous study has revealed that regions related to wakefulness, emotion, attention, worry, and sensory motor showed increased functional connectivity with each other when studying the whole‐brain functional connectivity in patients with primary insomnia (Li et al., 2017). The increased DAN‐FPC‐positive connectivity may provide evidence for the hyperarousal model of insomnia (Bonnet & Arand, 2010) at the connectivity level. Moreover, the functional connectivity between these two task‐activated networks was significantly correlated with RT of PVT in insomniacs. PVT is one of the leading assays of sustained attention in the sleep‐related research and is highly sensitive to the effect of sleep loss (Deurveilher, Bush, Rusak, Eskes, & Semba, 2015). Studies in rats of chronic sleep restriction or sleep deprivation and in patients with sleep–wake disorders or obstructive sleep apnea showed that poorer PVT performance was associated with more sleep loss and excessive daytime sleepiness (Batool‐Anwar et al., 2014; Deurveilher et al., 2015; Dinges et al., 1997; Doran, Van Dongen, & Dinges, 2001; Oonk, Davis, Krueger, Wisor, & Van Dongen, 2015; Thomann, Baumann, Landolt, & Werth, 2014). Insomniacs often complain about insufficient sleep and sleepiness in the daytime that make daytime alertness and sustained attention impaired. These impairments of alertness and sustained attention may be resulted from the abnormal DAN‐PFC functional connectivity.

Importantly, results showed that strength of aDMN‐pDMN functional connectivity was significantly decreased in insomniacs compared with HGSs. In contrast to DAN and FPC, DMN is considered as the task‐deactivated network, consisting of the medial prefrontal cortex, posterior cingulate cortex /precuneus, inferior parietal lobe, lateral temporal cortex, and hippocampal formation. Several studies have showed that DMN was associated with many psychological functions, including self‐referential mental activity, mind wandering, daydreaming, autobiographical memory retrieval, and future envisioning, which may be disrupted by certain disorders (Dai et al., 2015; Fox et al., 2005; Gusnard, Akbudak, Shulman, & Raichle, 2001; Mason et al., 2007; Raichle, 2011; Spreng, Stevens, Chamberlain, Gilmore, & Schacter, 2010; Uddin, Clare Kelly, Biswal, Xavier Castellanos, & Milham, 2009; Xu, Yuan, & Lei, 2016). Based on an enormous body of literature in which DMN is further divided into two subsystems: anterior default mode network (aDMN) and posterior default mode network (pDMN) (Damoiseaux et al., 2007; Lei et al., 2013, 2014), an interesting topic focused on the aDMN‐pDMN connectivity in insomniacs had attracted broad attention. Dai et al. (2015) found that primary insomnia patients showed decreased functional connectivity among the subregions of DMN. Otherwise, insomniacs may suffer reduced sleep duration, which resembles sleep deprivation. De Havas, Parimal, Soon, and Chee (2012) found that sleep deprivation was associated with significantly decreased functional connectivity within the DMN (De Havas et al., 2012). Similarly, another study revealed that DMN was splitted into two modules after sleep deprivation, and larger degree of decreased connectivity between these two modules was accompanied by worse mood (Wang, Liu, Hitchman, & Lei, 2015).

The current study did not use the seed‐based approach to calculate the functional connectivity, but in a way of large‐scale brain networks. We acquired two subsystems of DMN: aDMN and pDMN by ICA method. When insomniacs are preparing for sleep in the bed, most of their attention was focused on self‐referential processing such as ruminations, worries, or future envisioning (Harvey, Tang, & Browning, 2005; Marques, Gomes, Clemente, Moutinho dos Santos, & Castelo‐Branco, 2015). These psychological processes are involved in the activation of some regions of DMN. Furthermore, it has been proposed that persistent arousal in insomnia may be due to the overactivity of some brain areas of DMN before or during sleep, and even during the period of wakefulness. Thus, the functional connectivity between DMN subsystems was decreased in insomniacs (Marques et al., 2015; Nofzinger, 2010).

Several limitations of the current study should be noted. First, the study lacked some scales for control group, which may limit the comparative analysis between two groups. However, we focused more upon the relationship between these scales' values and functional connectivity of large‐scale brain networks in patients, so it did not influence the results of patients. Second, some insomniacs had taken medicine, which may partially affect the results. To make the results of this study convincing, these insomniacs have been prohibited taking medicine 24 hr before the experiment. Third, we did not have the polysomnography data of patients, but all of these clinical insomniacs were diagnosed by two psychiatrists according to the International Classification of Sleep Disorders: Diagnostic and Coding Manual, 3rd ed., and measured by the PSQI questionnaire. Finally, our cross‐sectional design did not allow us to precisely assess the change in behavioral performance and functional connectivities of resting‐state brain networks. To conclude, our study suggested that patients with ID exhibited the aberrant resting‐state functional connectivity of triple network model, involving DMN, DAN, and FPC, and provided novel evidence about the significant association between increased DAN‐FPC functional connectivity and PVT performance in patients with ID, which may be the underlying neural mechanism for the hyperarousal hypothesis in insomnia.

Thus, the results supported the hyperarousal hypothesis of insomnia, emphasizing the cognitive hyperarousal with more self‐referential processing such as rumination, worry, and intrusive thoughts (Perlis, Shaw, Cano, & Espie, 2011; Riemann et al., 2010). Furthermore, this study can provide an enlightenment to treat insomnia in clinical application through enhancing the insomniacs' thought control ability by cognitive behavioral therapy (Hood, Rogojanski, & Moss, 2014; Okajima, Nakajima, Ochi, & Inoue, 2017) and adjusting the functional connectivity by neurofeedback therapy (Arns & Kenemans, 2014; Marzbani, Marateb, & Mansourian, 2016).

## CONFLICT OF INTEREST

None declared.
